# A case of epicardial connection across the mitral isthmus line revealed by only endocardial mapping

**DOI:** 10.1016/j.hrcr.2024.04.015

**Published:** 2024-05-09

**Authors:** Satoko Shiomi, Seigo Yamashita, Kenichi Tokutake, Michifumi Tokuda, Michihiro Yoshimura, Teiichi Yamane

**Affiliations:** Division of Cardiology, Department of Internal Medicine, The Jikei University School of Medicine, Tokyo, Japan

**Keywords:** Catheter ablation, Epicardial connection, High resolution mapping, Marshall bundle, Perimitral atrial tachycardia

**Key Teaching Points**

• Although the assessment of epicardial potentials in the vein of Marshall (VOM) is useful for the diagnosis of perimitral atrial tachycardia via epicardial connection, it is challenging in cases with poor VOM.
• We were able to distinguish the intramural or epicardial potentials from the endocardial potentials by using single extra-atrial stimulation methods and to notice the super-delayed potentials in the left pulmonary vein (PV) carina conducted via the epi-endocardial connection.
• The interruption of epicardial conduction could be confirmed during radiofrequency applications at the posterior side of the mitral isthmus line upstream of the Marshall bundle in real time by monitoring the super-delayed potentials recorded at the left superior PV antrum.

## Introduction

Perimitral atrial tachycardia (AT) via epicardial connections sometimes occurs after mitral isthmus (MI) line ablation, and the epicardial connection can be evaluated from the vein of Marshall (VOM). We herein report a macroreentrant AT involving the epicardial fiber in a patient with poor VOM. We succeeded in distinguishing epicardial signals by endocardial mapping alone using an atrial extrastimulus method, and were able to confirm interruption of epicardial connection during catheter ablation in real time.

## Case report

We encountered a male patient in his 70s who was referred for a third catheter ablation owing to AT that occurred after 2 previous atrial fibrillation (AF)/AT ablation procedures. In addition to pulmonary vein (PV) isolation, the MI line and cavotricuspid isthmus line were created in the initial procedure for persistent AF, and ridge-related AT and perimitral AT were successfully treated in the second procedure. Informed consent was obtained from the patient for publication of this case report.

**Figure 1:**
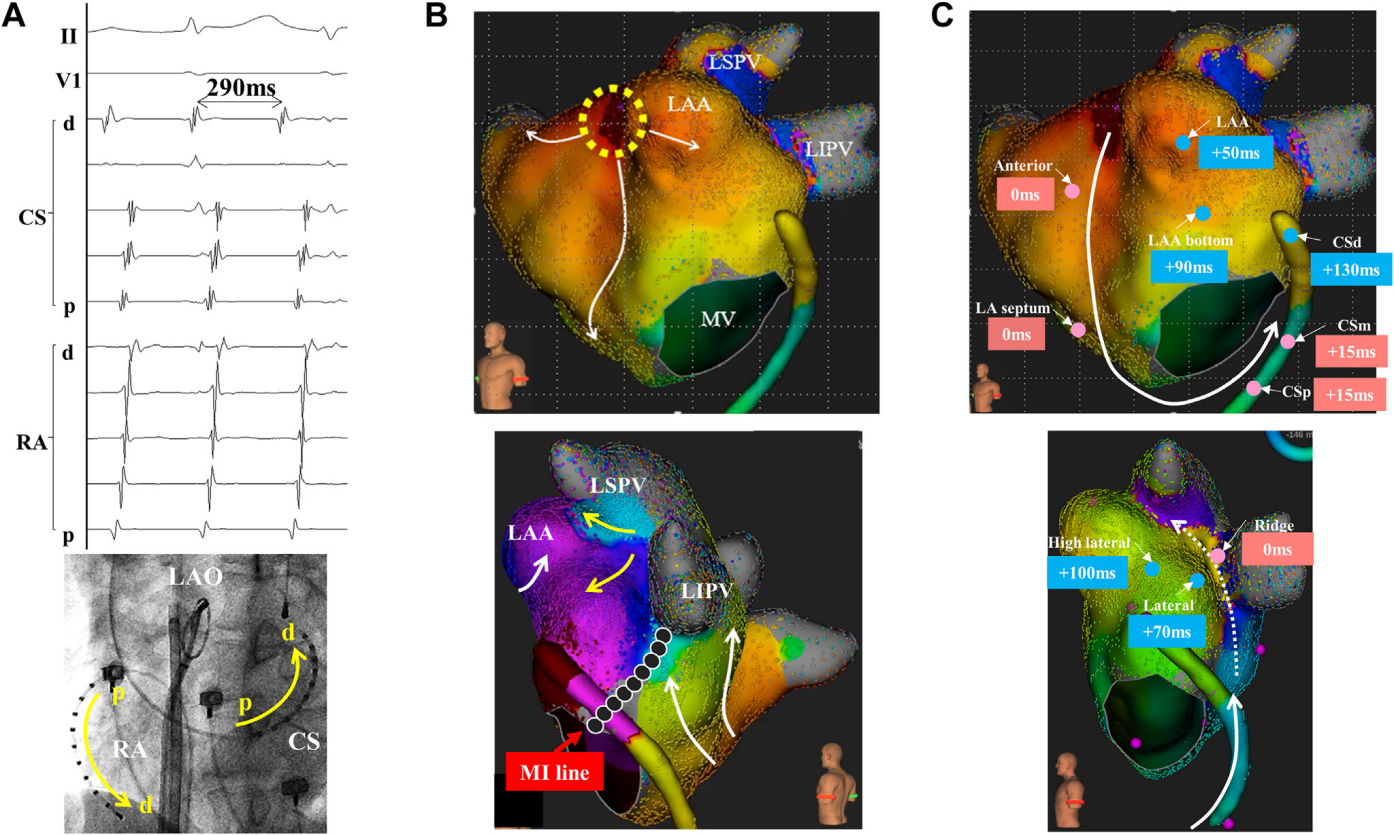
**A:** Clinical atrial tachycardia (AT) with a cycle length (CL) of 290 ms. **B:** A propagation map using Rhythmia (Boston Scientific, Natick, MA) showed a focal pattern originating from the left atrial appendage ostium (LAAos; *yellow dotted area*) with a endocardial conduction block on the previous mitral isthmus (MI) line (*black dots*), and then isolated delayed activation was observed from the carina and ridge (*yellow arrow*). **C:** The postpacing interval was consistent with tachycardia CL at the left atrial (LA) anterior wall, septum, proximal/mid coronary sinus (CS), and ridge but not at the LAAos, distal CS, or LA lateral wall, which indicated macroreentry AT via epicardial conduction along the Marshall bundle (*white dotted line*). d = distal; LAO = left anterior oblique; LIPV = left inferior pulmonary vein; LSPV = left superior pulmonary vein; MV = mitral valve; p = proximal; RA = right atrium.

We started the third procedure under sinus rhythm, and the absence of PV reconnection and establishment of an MI line with bidirectional block using differential pacing methods on the coronary sinus (CS) were confirmed initially. Burst pacing from the proximal CS induced clinical AT with a cycle length (CL) of 290 ms (Figure 1A). High-resolution mapping using the Rhythmia system (Boston Scientific, Natick, MA) demonstrated a focal AT pattern originating from the ostium of the left atrial appendage (LAA), but isolated delayed activation was observed around the ridge and carina (Figure 1B). The “LUMIPOINT” software revealed continuous activation at the ridge with transient interruptions on the MI line and top of the ridge (Supplemental Video 1), and total activation time along the perimitral circuit was 25 ms short of AT CL. Postpacing intervals after entrainment pacing at multiple sites along the mitral annulus and ridge were almost consistent with AT CL (Figure 1C), while postpacing interval at the site adjacent to the ridge was much longer than AT CL, which strongly suggested perimitral AT via the epicardial connection. Although we attempted to insert a microcatheter (EP Skinny; Kaneka Medix, Tokyo, Japan) into the VOM to evaluate the epicardial connection, the VOM was too small to be inserted distally. Then, entrainment pacing from the VOM terminated AT, and AT could not be induced anymore.

**Figure 2:**
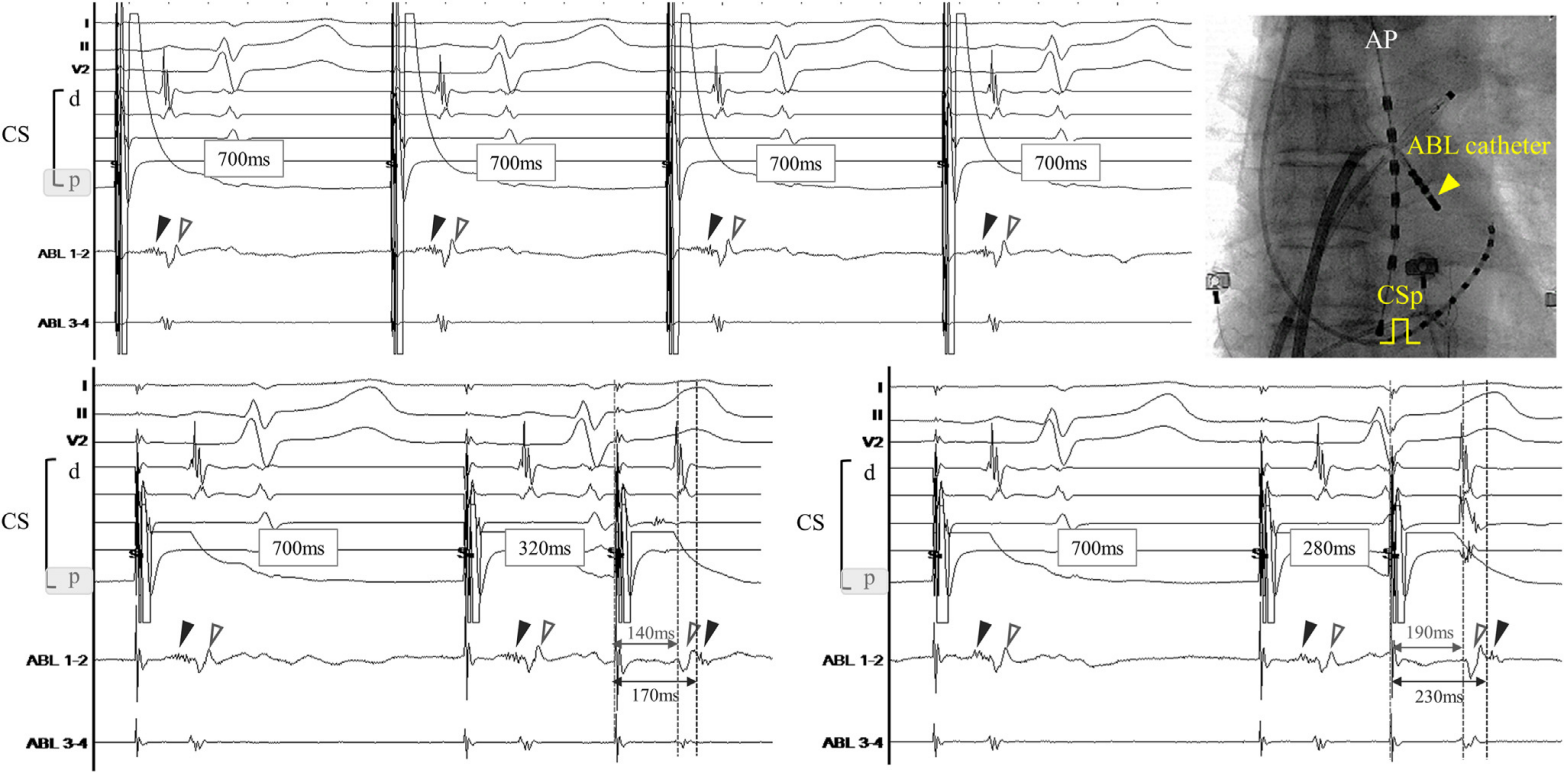
Upper: A fragmented potential with a small amplitude (*black triangle*) and a dull potential with large amplitude (*white triangle*) were simultaneously recorded at the ridge during proximal coronary sinus (CS) pacing. Lower left: These 2 potentials reversed when the interval of single extrastimuli from the proximal CS was shortened from 340 to 320 ms, and these phenomena were observed reproducibly and pronounced with shortening of the single extrastimulus interval to 280 ms (Lower right). ABL = ablation; AP = anterior-posterior; d = distal; p = proximal.

**Figure 3:**
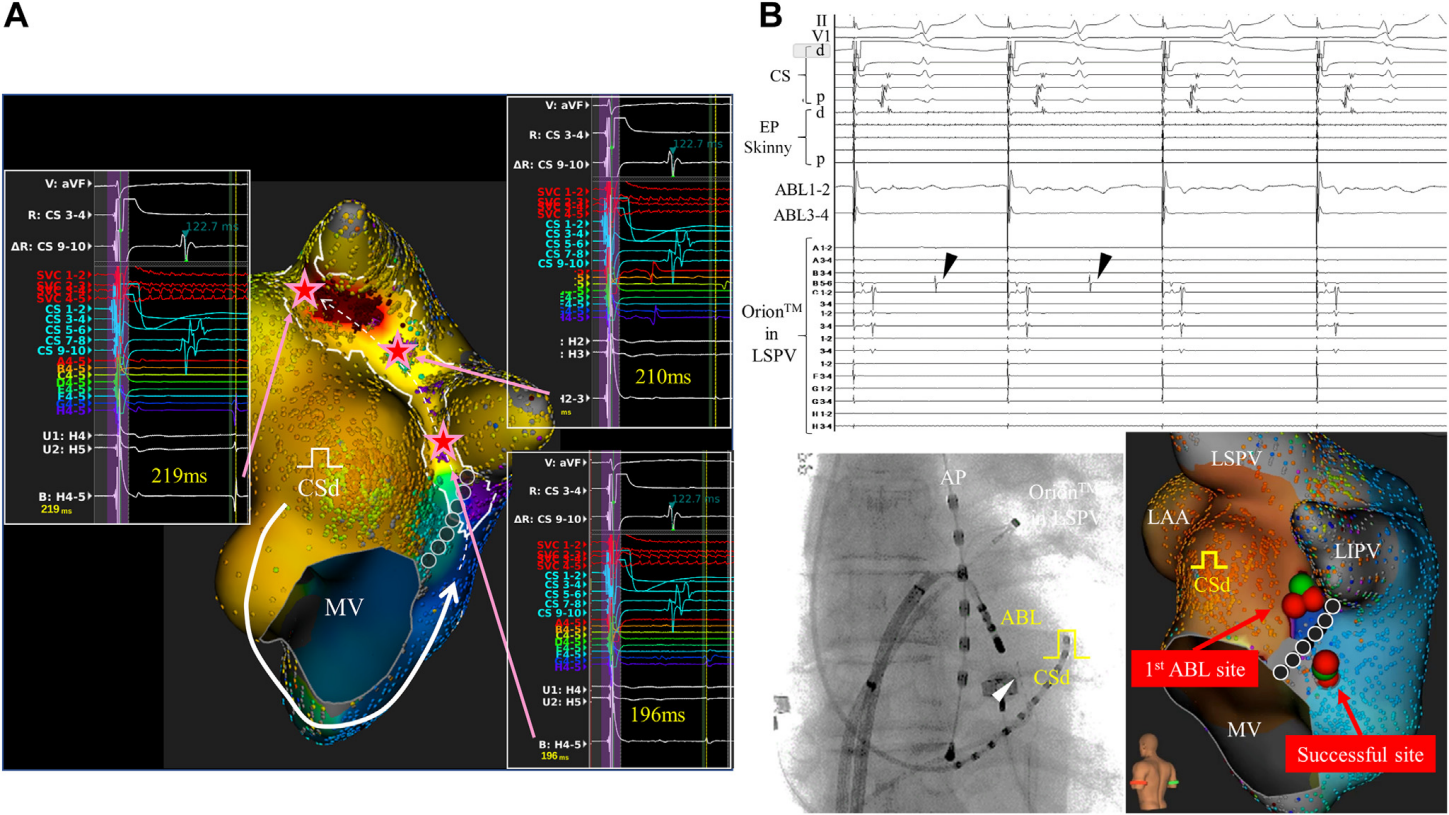
A: The LUMIPOINT Complex Activation software highlighted the fractionated signals around the ridge and left pulmonary vein (PV) carina, and super-delayed and small potentials propagating from the ridge to left PV carina could be confirmed during distal coronary sinus (CS) pacing. **B:** The super-delayed potentials (*black triangle*) on the Orion catheter (Boston Scientific, Natick, MA) placed at the left superior PV antrum disappeared during radiofrequency application at the posterior side of the mitral isthmus line upstream of the Marshall bundle. Of note, no potential was recorded on the ablation catheter at the successful site. The white triangle indicates the micro mapping catheter (EP Skinny) within the ostial vein of Marshall. ABL = ablation; AP = anterior-posterior; d = distal; LAA = left atrial appendage; LIPV = left inferior pulmonary vein; LSPV = left superior pulmonary vein; MV = mitral valve; p = proximal.

Because the epicardial side could not be directly evaluated, mapping was performed on the endocardial side. A fragmented potential with a small amplitude and dull potential with a large amplitude were simultaneously recorded at the ridge. To discriminate between these potentials, single extrastimuli from the proximal CS were performed. Interestingly, the fragmented potential showed a decremental property with shortening of the extrastimulus coupling interval, and finally these 2 potentials reversed (Figure 2). These findings suggest that these 2 potentials are derived from different myocardial fibers with different conduction properties. In addition, the activation timing of the dull potential was the same as that of the left atrial muscle represented on the distal CS; therefore, the fragmented potential was thought to represent activation of the intramural or epicardial myocardial fiber. A propagation map was created during distal CS pacing to evaluate detailed activation of the epicardial connection. As shown in Figure 3A, super-delayed and small potentials activating from the ridge to left PV carina with fragmented potentials were highlighted by LUMIPOINT Complex Activation software, propagating from the left atrial (LA) posterior wall with transient interruption on the MI line. Radiofrequency (RF) was applied using a 4-mm-tip irrigated ablation catheter (INTELLANAV STABLEPOINT; Boston Scientific) at the ridge with delayed fragmented potential under the guidance of PV potentials on the Orion mapping catheter (Boston Scientific) placed at the left superior PV antrum. The local delayed potential on the ridge and super-delayed potentials on the Orion catheter were gradually prolonged and disappeared but reconnected immediately. Since the epicardial connection could not be disconnected by several RF applications in the same area, we targeted the posterior side of the MI line upstream of the Marshall bundle, and super-delayed potentials recorded at the left superior PV antrum were eliminated during RF application (Figure 3B). The final propagation map during distal CS pacing demonstrated no fragmented potentials along the ridge and no potential at the left carina, which indicated the elimination of epicardial conduction and establishment of the MI block line. The patient was free from AF/AT recurrence after 1 year of follow-up.

## Discussion

The confirmation of a complete MI block line is still challenging in some cases owing to pseudo block, even though many diagnostic methods have been previously proposed.^1^ Persistent epicardial conduction across the MI line is a particularly important cause of pseudo block, and electrophysiological maneuvers, including the assessment of CS activation forms, differential pacing, and double potentials along the line, cannot reveal pseudo block in such cases. To avoid this kind of misdiagnosis, detailed high-density mappings under atrial pacing from both sides of the MI line are essential,^2^ and the assessment of epicardial potentials in the VOM can also be useful.^3^ In the present case, perimitral AT via epicardial connection could be presumed by the entrainment pacing maneuver; however, a direct assessment of epicardial potentials was impossible because of poor VOM. Furthermore, the unidirectional block of the endoepicardial connection with super-delayed conduction made it more difficult to identify epicardial conduction. We were able to distinguish the intramural or epicardial potentials using single extra-atrial stimulation methods and to notice the super-delayed potentials in the left PV carina. Multiple RF applications targeting the presumed epi-endo connection at the ridge were ineffective; however, PV potentials via epi-endo connection disappeared in real time during RF applications upstream of the VOM and fragmented signals on the ridge were simultaneously eliminated, suggesting that the fragmented potentials recorded on the ridge might have represented intramural or epicardial potentials.

In the present case, the following 2 types of epicardial connections were considered: (1) the Marshall bundle connected to the LAA ostium with a branch to the left PV and ridge, or (2) 2 different epicardial connections in the Marshall bundle connected to the left PV and ridge, and another epicardial fiber between the left superior PV and LAA ostium. Although epicardial connections from the Marshall bundle to the left PV and ridge have been clinically reported,^2^, ^3^, ^4^ we could not prove an accurate circuit because of the lack of entrainment pacing at the left PV carina. However, these possible epicardial connections were ultimately incapacitated by catheter ablation upstream of the Marshall bundle.

## Conclusion

We experienced a macroreentrant AT involving the epicardial fiber in a patient with poor VOM. This is the first report to discriminate intramural or epicardial potentials by endocardial mapping alone using atrial extrastimuli methods, and interruption of epicardial conduction could be confirmed during RF applications in real time.

## Disclosures

SS: None; SY: None; KT: None; MT: None; MY and TY: There is no COI to disclose directly related to this case.

## Appendix Supplementary Data

Supplemental Video 1 The LUMIPOINT Propagation revealed continuous activation along the ridge with transient interruptions on the previous mitral isthmus (MI) line and top of the ridge.
